# Avoiding Crowded Places During COVID-19: Common Sense or a Complex Strategic Decision?

**DOI:** 10.3389/fpsyg.2021.700640

**Published:** 2021-11-15

**Authors:** Martijn Stroom, Piet Eichholtz, Nils Kok

**Affiliations:** Department of Finance, School of Business and Economics, Maastricht University, Maastricht, Netherlands

**Keywords:** behavioral science, public health communication, collective human behavior, human decision science, cognitive psychology, health psychology, COVID-19

## Abstract

**Introduction:** Following a period of strict lockdowns during the COVID-19 pandemic, most countries introduced policies in which citizens were expected to avoid crowded places using common sense, as advised by the WHO. We argue that the ambiguity in the recommendation to “avoid crowded places” implicitly forces individuals to make a complex strategic decision.

**Methods:** Using a Dutch representative sample of 1,048 participants [42% male, mean age=43.78years (SD=12.53), we examine the effect of context on the decision to visit a hypothetical recreational hotspot under the policy recommendation to “avoid crowded places.” We randomize four levels of context on the crowdedness “on the streets” (no context, low, medium, and high context). Subsequently, participants are asked to estimate the percentage of others going out in the same situation. Finally, we assess the impact of a selection of personal characteristics on the likelihood of visiting a crowded place.

**Results:** Respondents are proportionally more likely to go in a low context and high context, compared to no context (diff=0.121, *p*<0.000, and diff=0.034, *p*<0.05, respectively) and middle context (diff=0.125, *p*<0.000, and diff=0.037, *p*<0.05, respectively). Low context information also decreases the expectation of others going out (−2.63%, *z*=4.68, *p*<0.000). High context information increases the expected percentage of others going out (significant only for medium to high context; 2.94%, *z*=7.34, *p*<0.001). Furthermore, we show that education, age, and health and risk attitude are all predictive of the likelihood to visit a crowded place, notwithstanding the context.

**Discussion:** Although there is a strong inclination to avoid crowded places during the COVID-19 pandemic (81%), we find two context-driven exceptions: when people expect to avoid crowded spots (in the “low” context, i.e., strategical decision-making) and when people expect others to go (social influence). The freedom provided by ambiguous public policy is implicitly asking more from the population than it initially seems. “Use your common sense” is often the accompanied advice, but our results show that more and better information concerning the context is essential to enable us to make an optimal decision for ourselves, and for society.

## Introduction

Since the outbreak of COVID-19, countries across the globe have attempted to find ways to contain the rapid spread of the virus. Following a period of strict lockdowns, most countries proceeded towards a policy in which citizens were expected to avoid crowded places, as advised by the WHO (World Health Organization, 2020). Limiting movement to local recreational hotspots as well as (inter)national holiday destinations is considered essential in combating the swift diffusion of COVID-19 infections. Even during the “second wave,” avoiding crowded places remains the cornerstone of worldwide policies (National Center for Immunization and Respiratory Diseases (NCIRD), 2020). Following policy advice, however, has proven to be more challenging for the population than initially expected. Popular recreation spots often remain well-visited and shopping centers are almost as crowded as they were a year ago, especially in large cities (BBC News, 2020). Over the Summer of 2020, news and social media showed crowded beaches and partying adolescents almost on a daily basis.

The increase in people visiting crowded places appears irrational from a health perspective, but might be less surprising than expected. Accurately assessing the risk of self-behavior proves to be hard, the urge to recreate seems to grow over time, and the duration of the current situation is testing the limits of human patience and self-control (Huremović, 2019). Moreover, what is considered “crowded?” This uncertainty increases the number of factors and potential outcomes individuals consider (Martínez-Marquina et al., 2019). Whereas recent research discusses theories explaining refusal to comply to COVID-19 restrictions (Demirtaş-Madran, 2021), little to no attention has yet been provided to the thought process that underlies the decision to leave the home, against most policy recommendations, and visit popular recreation areas or crowded shopping streets. Understanding the human thought process from a behavioral perspective, beyond merely labelling behavior to be defiant, will help governments to be more effective in implementing COVID-19-related policies.

This paper investigates the decision of individuals whether or not to avoid crowded places, in a representative sample of the Dutch population, aiming to identify decisive factors underlying this choice. We expect the dependency of the outcome of one’s own action on the (unobservable) actions of others to dominate the decision-making process. Therefore, we specifically examine the effect of social context on the decision to visit a crowded place. We hypothesize that providing information on the crowdedness in general will be crucial in the decision of individuals to go out. Specifically, strategic decision making will motivate most people to go out when they expect others to stay home, inevitably leading to escalation and subsequent failure to avoid crowdedness. Similarly, explicit escalation will follow once the general expectations are that most people will go. The aim of this paper is threefold: First, we discuss which decisional processes and conflicts arise due to the ambiguity in the current policy, through the lens of a theoretical framework. Second, using experimental data we demonstrate that (social) context significantly influences the decision-making process of individuals. Finally, we show which personal characteristics have an effect on the decision not to go and how this differs per context. The latter also allows us to draw conclusions on which decisional processes drive the behavior of individuals.

## Theoretical Framework

### The Need to Leave

Psychology is unanimous about the inherent human need for social interaction. Baumeister and Leary claim that the need for frequent interactions with others is a necessity for emotional stability (Baumeister and Leary, 1995). We desire both close individual contact, as well as the ability to function in social groups (Bugental, 2000). Not meeting these requirements leads to invasive negative effects, including, but not limited to physical health and mental well-being (Baumeister and Leary, 1995). Poor social relationships are estimated to have an effect on mortality similar to smoking 15 cigarettes daily (Holt-Lunstad and Smith, 2012). Recently, a review by Serafini et al. (2020) confirmed the negative impact that frustration, boredom, and disabling loneliness have on (mental) health, specifically following the current COVID-19 pandemic. Social support is one of two main protective factors to avoid mental health issues during this crisis.

The COVID-19 pandemic threatens the ability to meet these basic social needs. This leads to a clear cognitive conflict: people are craving for social contacts, regardless of rapidly rising contaminations with the virus. Using the health belief model framework (HBM; Champion and Skinner, 2008), even without a change in susceptibility or severity of an infection, the downside (i.e., barriers) of staying home slowly starts to compete with the benefits. Such a cognitive conflict, better known as cognitive dissonance, can be dealt with in two ways: changing the behavior or changing the reasoning (Festinger, 1957). From a societal perspective, reasoning in favor of keeping distance at all cost, taking no risk, would be preferred. However, the need to socially interact is growing: we observe society-wide violations of the universal policy discouraging social interactions (BBC News, 2020). Going out and being amongst people (albeit within the set regulations) is gaining traction over the safer, more certain option to stay at home to avoid health risks.

### Strategic Decision-Making

Acknowledging that the motivation to recreate is strong, the actual decision to “go” or “not to go” to a crowded location depends on the information that is available to the individual at the moment of making the decision. The recommendation to avoid crowded areas is not black or white, and it requires each individual to estimate which spots are considered popular at a given point in time. Although we can assume that every community has a relatively objective view of what is considered a crowded area, the recommendation to avoid these areas implicitly requires an individual to correct for the current situation: how busy will a potentially crowded area be at the moment that I intend to visit it? The degree of crowdedness is determined by the number of people considering to go, their thought processes, and their final decision.

We argue that the seemingly simple choice to visit or avoid a crowded place implicitly involves at least three complex strategic decision-making aspects. First, the choice generally draws parallels with the tragedy of the commons. Hardin describes the tragedy in which a shared yet unregulated common good (in this case, the location for recreation), is spoiled by society because each individual acts according to his or her self-interest, so “depleting” the common good (Hardin, 1968). The similarity lies especially in the fact that collective cooperation would retain the common good, but the individual interest conflicts with the collective maintenance of the good. In this situation, each individual selfishly wants to be in the minority group that visits the recreation area. When too many people act selfishly, the area becomes too crowded and the location no longer meets the “avoid crowded areas” requirements to minimize the spread of COVID-19 infections. In a worst-case scenario, “depletion of the good” could be the closure of the area for recreation, or even reimplementation of a full lockdown.

Second, and more formally, the dependency of each individual’s outcome on the choice of the remainder of the population closely resembles the classic game theoretical prisoner’s dilemma: going out will lead to a positive outcome if the majority of the population stays away, and only leads to a negative outcome if the majority of the population goes. This dilemma shows us that staying home is not a Nash equilibrium (e.g., an outcome of a decision in which no player has an incentive to deviate from his strategy; Nash, 1950). If everybody stays at home, each individual can improve his or her personal situation by going out. Going out, however, could be considered Nash equilibrium: when everybody is going out, staying at home would not improve somebody’s personal situation, when they would be the only person at home. Note that we assume that staying at home while everybody else is recreating comes at a (small) disutility, based on the fear of missing out (Przybylski et al., 2013) and not being able to meet the social craving. This makes the decision process oddly circular, and the outcome of the process depends heavily on the moment each individual breaches this circle.

Therefore, third, the decision process to optimize the outcome of the decision concerns *k*-level thinking and cognitive hierarchy theory (Stahl, 1993; Camerer et al., 2004). The core of this theory is that a person will determine strategy depending on the likely actions of others. The levels refer to the reasoning level someone expects the others to have, or “depth.” For instance, level 0 thinkers are considered non-strategic, choosing at random. Level 1 thinkers assume a majority of level 0 thinkers, and will strategically choose considering a random distribution of level 0 thinkers’ decision. Level 2 thinkers will, at their turn, assume a majority of level 1 thinkers, and so forth. In our example, we could hypothetically assume that level 0 thinkers “naively” stay away from a recreation area. As such, level 1 thinkers would come to the conclusion to go as the area will not be crowded. Consequently, level 2 thinkers stay away again, and so forth. The *k*-level framework states that each person believes to be at the highest level of thinking, with everyone else below that level, giving this person the unique advantage to best adopt a strategy. In reality however, the average population hardly seems to reach level 2 (Camerer et al., 2004; Ho and Su, 2013). The implications of the decision to leave home and visit a crowded location during the pandemic are crucial, since citizens likely aim to anticipate the behavior of the majority. When most people are at the same (fairly) low reasoning level, but believe they are “outsmarting” their fellow citizens, the chances of an unexpectedly crowded recreation area become very high. Ironically, even when effort is exerted to outsmart the majority and recreate when the majority stays home (thus intending to meet the policy requirements), the implications of cognitive hierarchy theory suggest an “accidental” or implicit escalation of crowdedness.

### Explicit Escalation

In addition to accidental escalation due to the application of wrong strategies by individual citizens, we must also consider explicit escalation, including conscious violation of policy recommendations. In this context, we consider the possibility of the proverbial sheep leaping the ditch: once a large enough group will ignore the policy recommendation, more will automatically follow. These people are, in contrary to the strategic thinkers, no longer intending to *avoid* crowded places. In pandemics this situation is called behavior contamination (Huremović, 2019). We discuss three types of violations, of which the latter two include cognitive processes that potentially influence the decision to ignore policy recommendations once violations by others are observed.

The first type of explicit violation is based on unrealistic optimism. In contradiction to the latter two types, unrealistic optimism is mostly independent of the behavior of others, as it pertains to the beliefs that the likelihood of something bad happening to you is smaller than it is in reality (Shepperd et al., 2015). Individuals might violate policy recommendations as a direct consequence of believing that a COVID-19 infection will not happen or harm them. This type of reasoning stems from both the desire to feel good, thus ignoring bad outcomes (Tyler and Rosier, 2009), as well as an overestimation of one’s personal characteristics compared to the general population (e.g., being healthier than others; Shepperd et al., 2002)). Although the effect of unrealistic optimism might be smaller for events happening beyond their own control (Klein and Helweg-Larsen, 2002), behavior due to unrealistic optimism is easily distinguishable from other “decision processes” in this situation: individuals will go independent of what other people do or think.

Second, a prevalent view in behavioral science is that these kinds of “deliberate” violations are the result of a loss of self-control or a dominating need to recreate (Huremović, 2019). Boredom and frustration resulting from the ongoing pandemic increases the vulnerability to violate the recommendations (Huremović, 2019; Brooks et al., 2020). Observing others ignoring the recommendations functions as a “broken window”: a small violation validates further violations, causing a spread through society (Keizer et al., 2008). This broken window effect, or bad apple effect, is strong even when just a small group of violators is observed (Rutte and Wilke, 1992; Kerr et al., 2009). In this context, seeing others doing something you would also like to do could provide enough of an incentive for citizens to join: why would you stay away if others do not?

Finally, an alternative view explaining why individuals would follow others to crowded places, despite regulations not to do so, involves how people deal with ambiguity. Besides uncertainty about other people’s decisions, we also need to consider that people are unsure about the definition of crowded places, or ambiguous regarding the interpretation of the recommendation. Should one take the recommendation as a strict rule, or interpret it more loosely? When ambiguity rises, we tend to use informational social influence to guide our decision (Deutsch and Gerard, 1955). This could lead to contradictions. For example, during the initial loose recommendation to wear face masks in public in the Netherlands, compared to the predominately mandatory use in the rest of Europe, 64% of Dutch citizens were in favor of making face masks mandatory. However, only 17% already wore them at that time (De Hond, 2020). Even when our personal opinion or preference might deviate, in practice we conform to (what we think is) the majority opinion in ambiguous situations (Allen, 1965). It is crucial to observe from this example that even in a contagious disease pandemic, in which rationally safety is absolutely not in numbers, other people’s behavior is still valued in situations of ambiguity. Observing others violating the recommendation to avoid crowded places could therefore be interpreted as the opinion of the majority, and act as information for one’s own judgement.

The distinction between the latter two views lies predominately in the underlying intention of the conscious violation. Under the former, the intention can be categorized as ill-intentioned, to the extent that there is no attempt to validate the violation of the recommendation at the start. This does not exclude the possibility that individuals will exhibit *post hoc* justification, fabricating reasons why the violation was acceptable or ethical, potentially in response to social disapproval (for instance, after not getting infected with the COVID-19 virus, people could argue that they were correctly assessing the risk ex-ante; (Curley et al., 1986; Haidt, 2001). Under the latter, the intention to deviate from the recommendation originates from confusion. We argue that this behavior reflects the inability to self-assess the ambiguity or uncertainty, leading to herd behavior (Muchnik et al., 2013). Distinguishing between these motivations might be possible by looking at the behavioral response to increasing social violations: for people motivated by ill-intention, going to a crowded place is linearly related to others going; for uncertainty-motivated people, this relationship might only be detrimental when a large enough group signals the “okay” to go. Regardless, however, both motives will inevitably lead to escalation.

## Materials and Methods

### Participants

We surveyed a panel of 1,048 individuals *via* Flycatcher, a well-regarded Dutch research organization with access to a high-quality panel used for top research (Bults et al., 2011; Peperkoorn et al., 2020), about their choice whether to go or not to go to a hypothetical recreational hotspot. Our randomly drawn sample from this panel was reimbursed for participation. This sample is heterogeneous in relevant personal characteristics, such as age (M=43.70, SD=12.52), education, gender (42% male), and occupation.^1^ We employ no explicit exclusion criteria, beyond restricting our sample to adults residing in the Netherlands. For an extended overview, see Table 1. This research was reviewed and approved by Maastricht University’s Ethical Review Committee Inner City Faculties (ERCIC_195_09_06_2020).

**Table 1:** Summary statistics.

Summary statistics					
		Mean	SD	Minimum	Maximum
Personal characteristics	Education category	2.46	0.63	1	3
	Male	42%			
	Age category	2.18	0.74	1	3
	Age	43.78	12.53	18	67
Risk attitude	General	5.09	1.92	1	10
	Social	5.23	1.95	1	10
	Health	3.87	2.02	1	9
Relatability	Similar	4.06	2.64	1	10
	Imaginable	6.28	2.55	1	10
COVID-19 exposure	Reported cases	0.0004188	0.0005787	0	0.0041598
	Hospital admissions	0.000108	0.0001425	0	0.001125
	Deceased	0.0000516	0.0000736	0	0.0005043
	*N*	927			

*Age categories are coded as 1 (18–30), 2 (31–50), and 3 (50+). Risk attitude is measure on an 11-point scale. For Health, a maximum risk score of 10 is never given. Relatability is measured on a 10-point scale. COVID-19 exposure measures are absolute values per 100 inhabitants. In our statistical analysis, these metrics are transformed logarithmically*.

**Table 2:** Pairwise correlations of independent variables.

Variables	(1) Education	(2) Age	(3) Similar relatability	(4) Imaginable relatability	(5) Reported cases	(6) Hospital admissions	(7) Deceased	(8) General risk attitude	(9) Social risk attitude
(1) Education	1.000								
(2) Age	−0.343^***^	1.000							
(3) Similar relatability	0.079	−0.162^***^	1.000						
(4) Imaginable relatability	0.165^***^	−0.173^***^	0.506^***^	1.000					
(5) Reported cases	0.068	−0.046	−0.052	0.017	1.000				
(6) Hospital admissions	0.083	−0.078	−0.050	0.006	0.958^***^	1.000			
(7) Deceased	0.089	−0.056	−0.034	0.013	0.930^***^	0.921^*^	1.000		
(8) General risk attitude	0.068	−0.013	0.124^**^	0.075	0.004	−0.023	0.017	1.000	
(9) Social risk attitude	0.071	0.052	0.049	0.076	0.022	−0.030	0.022	0.478^***^	1.000
(10) Health risk attitude	0.020	0.013	0.103	0.032	0.030	−0.008	0.033	0.508^***^	0.372^***^

*For this table, education and age are not transformed to categories. Education is on a 0 to 11 scale. All COVID-19 exposure (5–7) measures are stated per 100 inhabitants, and transformed to natural logarithm due to the skewed nature of the distributions. Note that he highest correlated factors were also included stepwise into the main model, to check for collinearity issues*.

* *p<0.05*;

** *p<0.01*;

*** *p<0.001*.

### Methods

Each respondent is asked to envision the following situation: *You live within 20 kilometers of a beach, river, forest, or lake. Under normal circumstances, you (and your household) will seek recreation, cooling and refreshing at this area when temperatures exceed 25 degrees Celsius. You do not have a comparable alternative at home*. We ask each participant to decide whether they will visit this area tomorrow, given that it will be 30 degrees Celsius, in five different situations. For the first two situations, the government’s recommendation differs: 1) “Stay home,” and 2) “Avoid crowded places.” For the remaining three conditions, we keep the government’s recommendation constant (“Avoid crowded places”), but we provide additional information about the situation on the streets: 3) “You see that it is still very quiet on the streets,” 4) “You see that the streets are slowly getting busier,” and 5) “You do not notice any difference in the degree of crowdedness as compared to last year.” We, respectively, label these levels of context as “Low,” “Medium,” and “High.” All scenarios are presented to the respondents in a randomized order.

We ask each respondent to state whether they will visit the recreation location in each of the scenarios by answering either “yes” or “no.” Next, for each randomly presented scenario, we ask participants what percentage of all other respondents they think will answer the previous question with “yes.” This percentage provides us with an indication of the expectation that participants have about the behavior of others.

Furthermore, we collect data *via* the Dutch Bureau of Statistics (CBS) on the local intensity of COVID-19 infections, hospitalizations, and COVID-19 related deaths. COVID-19 exposure is estimated using official government data matched to each individual on geographical location due to the fact that testing was severely limited until 6weeks before the experiment. Using public data, we avoid the subjective estimation that people ‘might’ have had it, influenced by individually factors such as different health beliefs. Using postcode estimation, personal characteristics do not influence the base-rate possibility of exposure to COVID-19 infections.^2^ These statistics are matched to each individual in the sample at the four-digit postal code level.

Additionally, we ask the respondents to state their general, social, and health-related risk attitudes on a Likert scale from 0 to 10 (Falk et al., 2016). The risk attitude questionnaire consists of validated questions, one per domain. For example, the general risk attitude question is formulated as follows: “How willing are you generally to take risk.” The answer scale for all three questions ranges from “totally not prepared to take any risk” to “very much prepared to take risk.” This questionnaire has proven to correlate heaviliy with more extensive and tedious risk attitude measures such as the lottery task^3^ ^4^.

Although the same recommendation of avoiding crowded places is a COVID-19 policy cornerstone throughout Europe (World Health Organization, 2020), the experienced situational context and timing of our survey is important to ensure external validity. The Dutch government issued an “intelligent lockdown” from March 15th until May 11th 2020. Until June 1st 2020, Dutch citizens were asked to stay home as much as possible. From June onwards, the recommendation to avoid crowded places became the main policy recommendation.^5^ Our respondents completed the survey during the first half of July 2020, 5–6weeks after the introduction of this recommendation. At this time, Netherlands had just over 51,000 confirmed cases of COVID-19, almost 12,000 hospitalizations, and just over 6,000 COVID-19-related deaths since the beginning of the outbreak (Statistieken over het Coronavirus en COVID-19, 2021). The timing of our data collection ensures that respondents had ample experience in dealing with the key policy recommendation and that the responses accurately reflected their current behavior. We furthermore consider it important that no new changes in the recommendations were announced at the time, such that the anticipation of new rules, or the signaling of a more liberal approach interfered with the validity of the response.

## Results

### The Effect of Context on Decision Making

**Figure 1:**
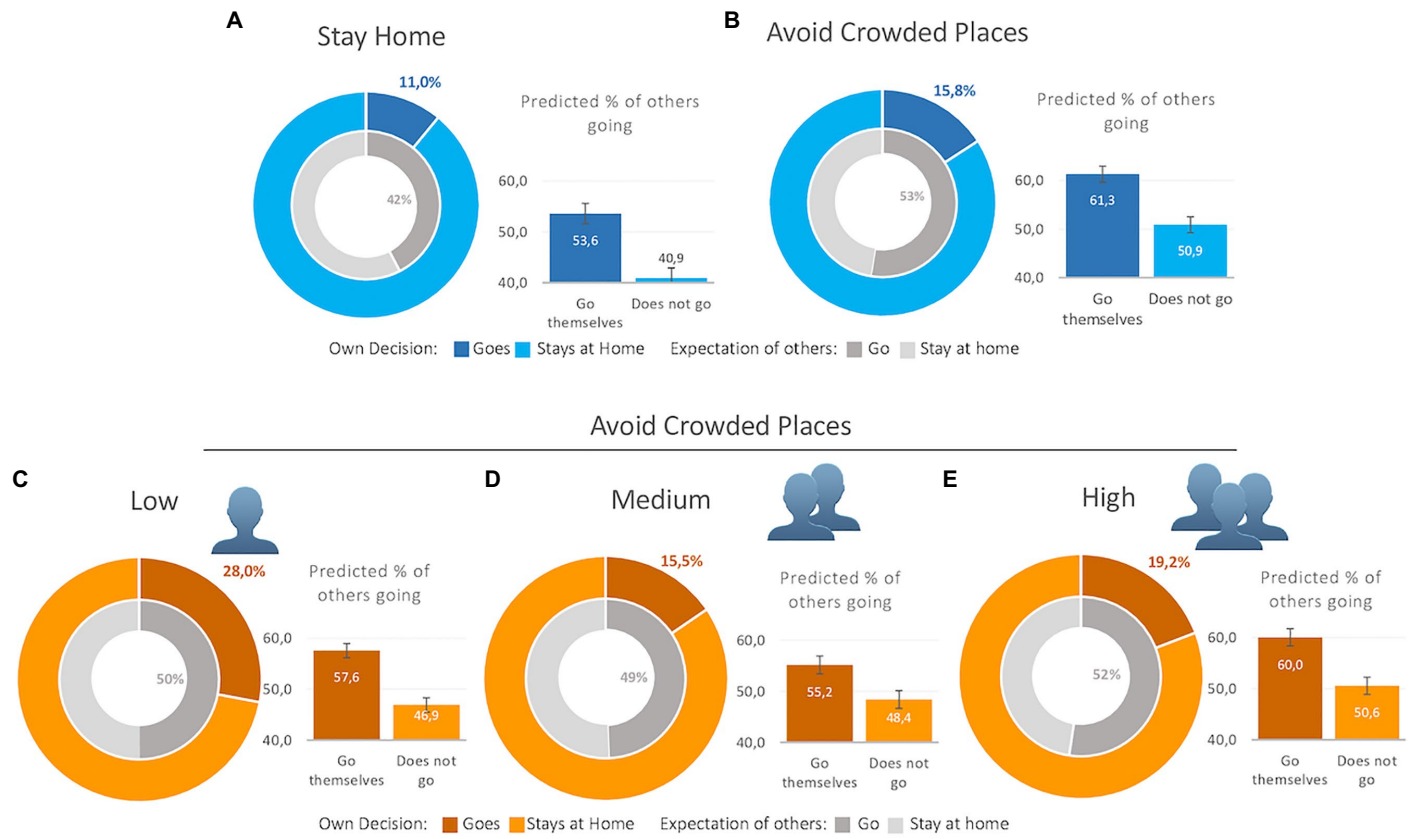
Statistics of intention to visit the crowded place. Note: The outer ring of the graph shows the percentage of respondents indicating to visit the crowded place, for each context. The inner ring shows the average expected percentage of others to visit the crowded place. The bar graph shows the same expected percentage of others to visit a crowded place, but split by respondents that indicate to go themselves versus respondents that indicate to stay at home. **(A)** Shows the metrics under the policy “stay home” without any further context. **(B)** Shows the same metrics but in the condition of “avoid crowded places.” **(C–E)** Show the graphics in this same condition, but each for a different level of crowdedness on the stress (low, medium, and high crowdedness, respectively). For an overview of the difference testing, see Tables 3 and 4.

Figure 1 presents whether or not respondents will visit a crowded area. In all scenarios, the vast majority of the respondents is not planning to go the recreation area. Although this appears encouraging for the policy objective to avoid crowded places, an average of 19% of all respondents across all five scenarios still decide to go.

Panel A shows the percentage of respondents indicating to go to the recreational area when the advice is to “stay home” (10.97%). The inner ring shows the average expected percentage of others to visit the crowded place (42%). Looking at the difference between the recommendation conditions “Stay home” (A) and “Avoid crowded places” (B), we observe a difference of just 5%. Finally, the bar graph shows the same expected percentage of others to visit a crowded place, but split by group of respondents that indicate to go themselves versus people that indicate to stay at home. For instance, for Panel A, people that go themselves predict that on average 53.61% of all other goes (SD=20.21), whereas the people that stay at home predict only 40.92% to go (SD=20.51). The difference between these two groups is statistically significant (see Table 3; *z*=−6.07, *p*<0.001).

**Table 3:** Statistics on the expectations of others going within each condition.

Predicted ratios of others’ going					
		Total	I will go	I will not go	*p*-value
Stay at Home	No context	42.31	40.92	53.61	0.00^***^
		(20.85)	(20.51)	(20.21)	
Avoid crowded places	No context	52.54	50.89	61.32	0.00^***^
		(19.93)	(19.78)	(18.41)	
	Low	49.90	46.93	57.58	0.00^***^
		(22.25)	(21.08)	(23.34)	
	Medium	49.44	48.40	55.19	0.00^***^
		(20.55)	(20.05)	(22.29)	
	High	52.38	50.57	60.03	0.00^***^
		(21.79)	(21.26)	(22.38)	
	*N*	1,048			

*First column shows the overall average predicted percentage of other’s going. The latter columns show the same statistic, split depending on the participants going themselves (“I will go”) versus staying home (“I will not go”). The size of these subgroups fluctuates per condition and context. Standard deviation in brackets. p-value based on non-parametric ranksum test*.

*** *p<0.001*.

When we add context about the level of crowdedness on the streets, we observe an additional increase in the number of respondents intending to leave the home. It is noticeable that providing a *clear* context about the crowdedness on the streets, regardless whether this is low (C) or high (E), causes a steep increase compared to the middle condition (D) and even no context (B). Panel A of Table 4 shows the results of a series of proportion test comparing the proportions per condition. It shows that likelihood of going out does not differ significantly between no context (B) and the middle condition (D) (diff=0.044, *p*=0.81). Both the low (C) and the high (E) condition differ significantly from both no context (B; diff=0.121, *p*<0.000, and diff=0.034, *p*<0.05, respectively) and the middle condition (D; diff=−0.125, *p*<0.000, and diff=0.037, *p*<0.05, respectively). Respondents are more likely to go to the area of recreation when they expect it to be quiet (overall most likely, even compared to the second most likely condition: high (E); diff=0.088, *p*<0.000). This is in line with both the official policy recommendation as well as strategic thinking. More surprising is that respondents are also more inclined to visit a popular area when they have reason to believe that it will be crowded at this location. This is directly opposite to the official policy recommendation, and not in line with game-theoretical predictions. This preliminary result suggests that respondents’ strategic thinking (in the low context) as well as social norms (in the high context) play a role in their decision whether to go, or not.

**Table 4:** Statistical testing of the difference between conditions: going versus not going.

	No context	Low	Medium
**Panel A – Proportion of going**			
Low	0.121^***^	0	
	(6.71)	(−)	
Medium	−0.004	−0.125^***^	0
	(−0.24)	(−6.94)	(−)
High	0.0338^*^	−0.088^***^	0.037^*^
	(2.01)	(−4.73)	(2.25)
N	1,048	1,048	1,048
**Panel B – Predicated percentage others’ going**			
Low	−2.63^***^	0	
	(−5.52)	(−)	
Medium	−3.10^***^	−0.45	0
	(−6.73)	(−0.91)	(−)
High	−0.16	2.48^***^	2.94^***^
	(−0.34)	(4.68)	(7.34)
*N*	1,048	1,048	1,048

*For both panels, the score is constructed such that the mean value of the row conditions is subtracted from the column conditions. Panel A shows the difference in proportions (proportion test) of people going out under different conditions. The outcome variables are binary such that 0=not going and 1=going. For example, people in the low context go on average 12.1% (0.280 minus.158, respectively) more often as compared to the no-context condition. Z-statistics in parentheses. Panel B shows the difference in predicted percentage (scored between 0 and 100) that people expect others to go. For example: people in the high condition expect that others will significantly go out more compared to both the low condition as well as the medium condition (2.48 and 2.94%, respectively). T-statistics in paratheses*.

* *p<0.05 and*

*** *p<0.001*.

We then investigate the estimation that respondents make about other’s behavior (Panel B, Table 4) using Wilcoxon rank-sum tests. We observe that respondents substantially and consistently overestimate the number of other people intending to go. Respondents expect, on average across all scenarios, that roughly 50% will decide to leave the home and recreate. Furthermore, the predicted percentages do significantly change between scenarios. We see significant changes in the prediction of other people’s behavior, indicative of the motivation of individuals to go themselves. For instance, introducing the “low crowdedness” context (C) compared to no context (B) almost doubles the number of respondents planning to go to the area of recreation (proportional increase of 12.1 percentage points, *z*=6.71, *p*<0.000), when the expected percentages of others going drops with 2.63% (*z*=4.68, *p*<0.000). Interestingly, moving from no context (B) or medium context (D) to high context (E), increases the proportion of people going with roughly 3.5 percentage points (3.4%, *z*=2.01, *p*=0.04; 3.7%, *z*=2.25, *p*=0.02, respectively), when also the prediction of others going increases (significant only for medium to high context; 2.94%, *z*=7.34, *p*<0.001). In general, introducing low context information increases going out whilst the expected percentage of others going out drops. Introducing high context information increases the likelihood of going out in conjunction with an increase in the expected percentage of others going out. However, the limited absolute value changes in the expectations about others indicate that the changes in one’s individual decision to go are not fully reflected in the prediction of other citizens’ behavior. In general, the expectations about others’ behavior are a lot more negative than one’s own behavior, and more negative than the behavior of the collective.

There is also a relationship between going out yourself and the expectations about others. Non-parametric Wilcoxon rank sum tests show consistently that, regardless of the scenario, respondents indicating a willingness to recreate themselves also predict a significantly higher number of people to make the same decision, compared to respondents indicating to stay home (all are significant for *p*<0.001; for an overview of these statistics, see Table 3). The prediction is significantly correlated with citizens’ own decision to go: for each percentage point increase in the prediction that others will go, the marginal effect of going themselves increases with an average of 0.3% (results are not presented in the table: ranging from 0.2 to 0.4%, *p*<0.001 throughout all contexts).

### Predictors

A key question is which factors are decisive for choosing to leave the home for recreation in each of these conditions. Table 5 investigates the role of personal characteristics in the choice for recreation per condition using a logit regression. The results show that education plays a key role in the decision to go, despite the regulation. The low education group turns out to be most likely to abide by the rules. The middle-educated category (post-secondary vocational degree, undergraduate education, or higher level of high school) are generally more inclined to go, compared to the low education group (post-secondary vocational education or lower level high school). The most highly educated respondents (undergraduate degree or higher) indicate an even higher willingness to go. The effect of education is most profound in the low (for middle education the marginal effect is 12.9%, *z*=2.43, *p*=0.015; for high education the marginal effect is 17.9%, *z*=3.29, *p*=0.001) and high context conditions (middle: 10.6%, *z*=2.41, *p*=0.016 and high: 11.0%, *z*=2.51, *p*=0.012, respectively). In the “medium” condition, we find no effect of education.

**Table 5:** Logit regressions: respondent characteristics and decision to go.

Marginal effects resulting from logit regressions					
		No context	Low	Medium	High
Education	Middle	1.073	1.129^*^	1.060	1.106^*^
		(1.85)	(2.43)	(1.43)	(2.41)
	High	1.095^*^	1.179^**^	1.069	1.110^*^
		(2.27)	(3.29)	(1.61)	(2.51)
	Female	0.984	1.006	0.963	0.995
		(−0.65)	(0.20)	(−1.57)	(−0.20)
Age	31–50	0.967	0.903^*^	0.990	0.991
		(−0.92)	(−2.27)	(−0.29)	(−0.25)
	Above 50	0.907^*^	0.791^***^	0.926^*^	0.919^*^
		(−2.57)	(−5.12)	(−2.12)	(−2.16)
Risk attitude	General	1.008	0.983	1.004	1.002
		(1.01)	(−1.76)	(0.55)	(0.21)
	Social	0.995	0.997	0.989	0.991
		(−0.70)	(−0.29)	(−1.61)	(−1.22)
	Health	1.016^*^	1.048^***^	1.020^**^	1.023^**^
		(2.57)	(5.54)	(2.96)	(3.16)
	Chi^2^	33.63	70.12	29.26	26.36
	N	964	964	964	964

*Education is relative to the baseline category “Lower education” and age is relative to the baseline category “30years or younger.” z-statistics in parentheses. Standard errors are clustered at the individual respondent level*.

* *p<0.05*;

** *p<0.01*;

*** *p<0.001*.

We also observe an effect for age, but not for gender. The effect for age is negative across all contexts. In the low crowdedness context, both age brackets have a significantly negative marginal effect (−9.7%, *z*=−2.27, *p*=0.023, and−20.9%, *z*=−5.12, *p*<0.001, respectively), whereas for all other contexts we observe older respondents (50+) to be less likely to visit the recreation location, compared to the 30-year and younger category. Interestingly, the impact of personal characteristics seems to diminish when the streets are getting busier: in the highly crowded context, both the significance as well as the strength of the effects of education and age decrease as compared to the “low” context.

The general and social risk attitudes do not have a significant influence on the decision of respondents. The degree to which respondents are willing to take risk with their own health, however, is important throughout all contexts. For each incremental increase of willingness to take risk on this domain, the probability that a respondent will go increases with 1.6% (*z*=2.57, *p*=0.01) to 4.8% (*z*=5.54, *p*<0.001) per context. This result implies that the decision to go depends more on respondent’s own health considerations than on the fear to contaminate others.

### Additional Explanatory Variables

#### Similarity and Imaginability

The hypothetical nature of self-reported vignette studies negatively affects their validity compared to actual behavioral measures (this is also referred to as the intention-behavior gap; Sheeran and Webb, 2016). The decision to go and visit a crowded place on a hot summer day will be influenced by the degree to which each respondent in our sample can relate to this specific scenario. For instance, a person living in a city center without a garden will likely better understand the motivation to go out of the house as compared to a person living in a rural area with big garden. To test whether these location-dependent characteristics influence the decision to go, we measure two additional indicators: level of similarity (e.g., to what extent the situation mimics their own situation) and the level of imaginability (e.g., to what extent are respondents able to imagine being in such a situation). For a summary of these metrics in our sample, see appendix Table 1.

We find that similarity increases the likelihood of visiting a crowded place. Panel A of Table 6 shows that for each increase on a similarity scale from 1 to 10, the marginal increase of going out ranges between 2.3 and 1.5% depending on the context (no context: *z*=5.25, *p*<0.001 and high context: *z*=−3.07, *p*<0.01, respectively). Beyond similarity, imaginability increases the probability of going out in the low context (1.8%, *z*=2.49, *p*<0.05) and high context (1.2%, *z*=2.03, *p*<0.05). In sum, both the similarity and imaginability of the situation increases the probability of visiting the recreation area, in most contexts.

**Table 6:** Logit regressions: location-dependent characteristics and decision to go.

		No context	Low	Medium	High
**Panel A**					
Relatability	Similar	1.024^***^	1.019^**^	1.023^***^	1.015^**^
		(5.25)	(3.22)	(4.90)	(3.07)
	Imaginable	1.004	1.018^*^	0.999	1.012^*^
		(0.80)	(2.49)	(−0.19)	(2.03)
	Chi^2^	33.63	70.12	29.26	26.36
	Controls	Yes	Yes	Yes	Yes
	N	964	964	964	964
**Panel B**					
COVID-19 exposure	Reported Cases	1.023	1.076	1.072	1.035
		(0.61)	(1.51)	(1.80)	(0.80)
	Hospital Admissions	0.973	0.972	0.948	0.963
		(−0.79)	(−0.63)	(−1.51)	(−0.96)
	Deceased	1.010	0.968	0.997	1.012
		(0.43)	(−1.10)	(−0.13)	(0.46)
	Chi^2^	32.26	62.90	29.09	28.19
	Controls	Yes	Yes	Yes	Yes
	N	840	840	840	840

*Panel A shows the marginal effect of the reliability measures on the decision to go. Panel B shows the marginal effect of COVID-19 different exposure indicators, using postal codes, on the decision to go. The measures are per 100 inhabitants, and transformed to natural logarithm due to a highly skewed distribution. Note that sample B consists of a smaller sample due to missing values in the COVID-19 database. Both panels are controlling for all personal characteristics presented in Table 1: education, gender, age, and risk attitude. z-statistics in parentheses. Standard errors are clustered at the individual respondent level*.

* *p<0.05*;

** *p<0.01*;

*** *p<0.001*.

#### COVID-19 Exposure

In order to generalize our results to other situations, and to show that policy and context drive the behavioral intensions that we observe, we assess the impact of COVID-19 exposure on the decision of our respondents to go. It is plausible that the survey participants experience the context we present to them in the light of their own experience of the COVID-19 threat. In order to investigate the robustness of our findings, we match all individual respondents to COVID-19 metrics that are publicly available through the Dutch Ministry of Public Health, using respondent postal codes (RIVM, 2020). Specifically, we standardize reported COVID-19 cases, hospital admissions, and COVID-19-related deaths such that for each postal code the value shows the ratio per 100 inhabitants.

Panel B of Table 6 shows the effects of local COVID-19 metrics on the decision to go, for each context. Due to the skewedness of all the metrics, we transformed the metrics using a natural logarithm. First, we find a marginally significant impact of the number of hospital admissions on the likelihood of going out at the medium level of context (−5.2%, *z*=−1.51, *p*<0.1). For all other levels, the number of hospital admissions and COVID-19-related deaths do not have an effect on the likelihood of going out. For the number of reported cases we find a marginally significant trend at the 10% significance level, having the opposite effect. Specifically, a larger number of reported cases suggests a higher likelihood of going out, only for the low and medium context, ranging from 7.6 to 7.2% increased likelihood (medium context: *z*=1.51, *p*<0.1 and low context: *z*=1.80, *p*<0.1, respectively).^6^

In summary, the influence of local COVID-19 exposure on our results, based on publicly available COVID-19 data, is weak and inconclusive. We observe an increased trend to recreate when there are more reported cases in the respondent’s postal code. However, this correlation could also be reversed in causality: more cases are reported because people tend to go and recreate. On the other hand, we find a comparable yet opposite likelihood of going for the local COVID-19 exposure of hospital admissions. The effects are concentrated exclusively in the “low” and in the “medium” condition, and they are only marginally significant.

Overall, given that both robustness analyses have an effect on the decision to go, we also added COVID-19 exposure measures, as well as the similarity and imaginability measures, as controls in the main regression of Table 5 (see Table 7). We observe only minor significance changes and no noteworthy changes in interpretation or direction of our previously discussed main results.

**Table 7:** Fully integrated logit regression: personal and location-dependent characteristics and decision to go.

Integrated model: marginal effects resulting from logit regressions					
		No context	Low	Medium	High
Education	Middle	1.072	1.126^*^	1.063	1.105^*^
		(1.88)	(2.23)	(1.47)	(2.30)
	High	1.084^*^	1.160^**^	1.056	1.096^*^
		(2.13)	(2.85)	(1.32)	(2.19)
	Female	1.002	1.024	0.975	1.013
		(0.09)	(0.70)	(−1.03)	(0.46)
Age	31–50	0.983	0.926	1.006	1.020
		(−0.44)	(−1.58)	(0.17)	(0.53)
	Above 50	0.922^*^	0.812^***^	0.938	0.945
		(−2.06)	(−4.23)	(−1.73)	(−1.47)
Risk attitude	General	1.004	0.981	1.003	0.999
		(0.45)	(−1.81)	(0.35)	(−0.10)
	Social	0.996	0.996	0.989	0.988
		(−0.62)	(−0.49)	(−1.51)	(−1.58)
	Health	1.015^*^	1.044^***^	1.015^*^	1.023^**^
		(2.33)	(4.85)	(2.37)	(3.14)
Relatability	Similar	1.024^***^	1.017^**^	1.023^***^	1.016^**^
		(4.90)	(2.63)	(4.70)	(3.00)
	Imaginable	1.002	1.014	0.998	1.010
		(0.39)	(1.84)	(−0.35)	(1.56)
COVID-19 exposure	Reported Cases	1.023	1.074	1.072	1.032
		(0.64)	(1.47)	(1.85)	(0.75)
	Hospital admissions	0.984	0.977	0.957	0.970
		(−0.51)	(−0.52)	(−1.28)	(−0.79)
	Deceased	1.005	0.968	0.992	1.010
		(0.24)	(−1.10)	(−0.33)	(0.40)
	Chi^2^	62.22	69.83	50.10	41.58
	N	840	840	840	840

*Education is relative to the baseline category “Lower education” and age is relative to the baseline category “30years or younger.” All COVID-19 exposure measures are stated per 100 inhabitants, and transformed to natural logarithm due to the skewed nature of the distributions. The sample is smaller compared to Table 3 due to missing values in the COVID-19 exposure data. z-statistics in parentheses. Standard errors are clustered at the individual respondent level*.

* *p<0.05*;

** *p<0.01*;

*** *p<0.001*.

## Discussion

Public health policies to contain COVID-19 infections are under heavy scrutiny. An important pillar of public policies in almost any country is the recommendation to “avoid crowded places.” This appears to be a straightforward message, but in reality, it is not, since it inevitably introduces considerations of other people’s expected actions in citizens’ own decision-making process. Although the results in this paper suggest that the majority of citizens adhere to the policy recommendation,^7^ the results also suggest that people are implicitly forced to make a correct estimation of the situation outside. This is not trivial to each individual. The results not only show that a vast majority of respondents is unable to make an accurate estimation about others’ behavior, but also that a wrong estimation could lead to a worsened outcome.

In line with the theoretical framework, providing information regarding the situation outside initially leads to a rational choice (e.g., when it is calm, the majority intends to go, and when it is reportedly getting crowded, more respondents intend to stay home). The strategic decision underpinning is most clearly illustrated when moving from “no” context to “low” context: a steep increase of people that go themselves, combined with a significant decrease in the expectation of others to go. However, once people know that it gets even more crowded outside (“medium” to “high”), respondents indicate a greater willingness to go out, combined with an increase in expectations about others going, possibly leading to an escalation in crowdedness. These observations seem to indicate behavior contamination (Huremović, 2019): the stronger the expectation that others will go, the more likely it is that people will go themselves (Keizer et al., 2008). Our results suggest this latter “explicit” violation of the public health regulation is more likely a result of using social cues for ambiguity management than a bad-apple effect. Comparing the behavioral trend from the “low” to “medium” and finally “high” context, we see that moving to more ambiguity (medium crowdedness context) leads to fewer people going (e.g., providing no context is almost identical to the medium context, strengthening the ambiguous interpretation of the medium context). Since we do not observe a linear increase in violation over intensifying crowdedness contexts, but a parabolic relation, we believe it is likely that we witness the social context as informative to behavior, instead of provoking “violating” behavior. Overall, both theoretical predications are supported: strategic decision making seems to motivate people to go out when they expect others to stay home, whereas explicit escalation follows once the general expectations are that more people will go out.

The heterogeneous effects of multiple predictors on the decision to go gives crucial hints on the motivation and underlying thought process per context. A key indicator is the effect of education on the low and high context suggests that educational background is more important in the rational or strategic (low context) decision, than in the escalation (high context). Thus, we conclude that in the low context situation, highly educated people act strategically and in the medium context the social norm is leading in coping with the ambiguity. In the high context, social norms lead to escalation. Second, overall, the willingness to take risk in the health domain is an important predictor to go out: the higher the willingness to take health risks, the higher the likelihood of going out. Interestingly, this effect is strongest in the low context condition. The marginal effect of the willingness to take risk in the health domain is almost double compared to the other conditions. We observe the same for age: older individuals are less likely to go out in general, but the effect is almost twice as big in the low context condition compared to all other conditions.^8^ Although our results do not imply causality, and must therefore be interpreted with caution, they are not contradicting our previous conclusion: in the low context, a strategic decision process underlines the decision to go. Education, health, and age weigh heavily in the ultimate decision. These factors weigh less strongly in the “high context” condition, where the decision to go is rather motivated by behavior contagion instead of individual considerations. In other words, in a “low context” situation, people decide themselves, in “high context,” others (at least partially) decide. Specifically, in line with the Health Belief Model (HBM; Champion and Skinner, 2008) it is likely that perceived susceptibility and severity of the infection are influenced by the social context. Seeing others go out, might signal that others estimate the severity lower than they themselves do, lowering the motivation to stay inside (leading to behavior contagion).

The context that is given to people in their decision-making process is thus detrimental, but does not have a uniformly positive effect. Additional relevant factors such as willingness to take risk with one’s own health and the similarity to one’s own situation all increase the likelihood to visit crowded places.

It is also evident that people underreact to the behavior of others. In general, we observe incorrect pessimism about other people’s behavior: across all conditions, people expect far more people to go than the collective intention to do so. However, individuals also underestimate the effect context has on others, even when it has a profound effect on our own behavior. In other words, when the context influences people to go, people underestimate the increase in crowdedness as a result of other people making the same judgement due to the same change in context. This causes an escalation in the “low” context: Although the crowdedness context signals a quiet situation at the location of recreation, people do not take into consideration that the majority will come to the same conclusion. As such, our findings result in the somewhat paradoxical prediction that it will be busiest in the low crowdedness context.

In conclusion, the main aim of this paper pertains to assessing the impact of an ambiguous policy to “avoid crowded areas,” leaving individuals to form expectations about the level of crowdedness themselves, without guidance on which information they can use to come to this assessment.^9^ We show that, providing individuals with an *ad-hoc* proxy for crowdedness of which the informational value is unclear, leads to suboptimal yet predictable thought processes and decisions. Specifically, we show that a considerable number of people think they are strategically avoiding crowded places when it is quiet outside, and follow the herd when it is busy.

### Limitations

We strive to identify how the current (Dutch) COVID-19 policy recommendations, combined with limited information availability, influences behavior of individuals. In doing so, we intentionally strike a balance between a rigid experimentally controlled design, and elicitation of real-life ambiguity that closely reflects the current situation that individuals find themselves in. Loosening the experimental controls often comes at the cost of increasing the likelihood of omitted variables. Below, we discuss three main limitations of this study.

First, to achieve real-life ambiguity, our experiment is intentionally ambiguous in two dimensions: the location of recreation and the level of crowdedness. The first ambiguity increases the probability that the participant empathizes with the hypothetical situation. Specifying the location would surely have increased uniformity in beliefs about the expectations of crowdedness, travelling factors, or density of the location (e.g., how crowded is a beach compared to a forest or city center?). We acknowledge that omitted variables directly related to the preferred location might influencing the decision. However, keeping the location as a general category increases the likelihood that participants are able to envision themselves in this hypothetical location, regardless of their personal preference. This means our results can be generalized. Indeed, respondents in the sample state that they are able to envision themselves in this situation (average imaginability score of more than 6 out of 10), even though respondents might not necessarily be in this situation (average similarity score is only 4 out of 10). The second ambiguity is on the degree of “crowdedness.” This is not stated as an objective measure, but as a subjective experience that depends on the interpretation of the participant. For example, “the streets are slowly getting busier” aims to elicit a general tendency of increasing crowdedness in the community, but could be influenced by the literal interpretation of what the individual considers “the streets” as well as “getting busier.” Moreover, we consider these conditions to be at least ordinal in our interpretation, but the proportional distance between these levels can only be assumed. We are therefore unable to exclude that, in both dimensions, the interpretation of the ambiguity may lead to other reasoning and thus other behavior than we anticipate. However, note that these ambiguities are present in real-life decisions as well. We argue that the value of generalizability (at least partially) compensates for these potential omitted influences.

Second, we take a wide variety of individual factors and traits into consideration, but must acknowledge that additional personal beliefs and traits might matter as well. Most profoundly, we explicitly do not discuss motives for individuals to stay at home throughout all conditions and contexts. For instance, Jeong et al. (2016) mention the most frequent reaction to a pandemic to be the uncontrollable fear for infection. Individuals that were exposed to infection are more likely to develop worries about their own health and infecting others. Especially pregnant women and parents with children are likely to develop such fears (Braunack-Mayer et al., 2013). By focusing on individuals that consider to go, we neglect motives to not go at all. Seeing that the majority in our sample chooses not to go out at all, we feel strongly that psychological factors such as pervasive anxiety and uncontrolled fear (Serafini et al., 2020), as well as individual self-efficacy and perceived benefits of staying at home (Health Belief Model; Champion and Skinner, 2008), are key drivers for this behavior. However, this paper does not focus on the decision to go at the extensive margin, and is therefore unable to explain key drivers not to go at all, regardless of social context. More extensive research should focus on including and identifying the crucial factors determining the absolute choice to stay home.

**Figure 2:**
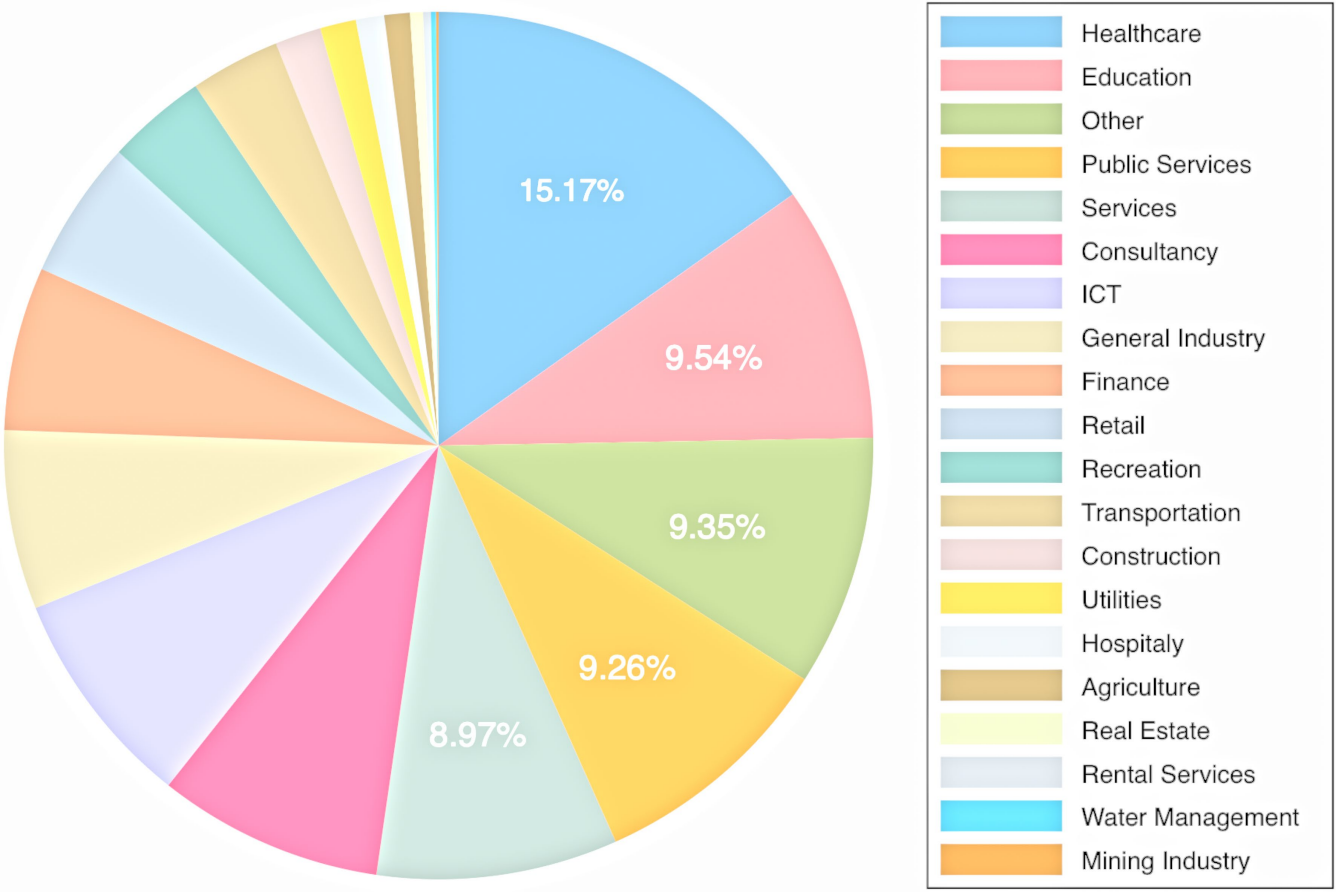
Distribution of occupations. Note: The graph includes the percentage of respondents for the five largest groups of professions in our sample, making up for more than half our sample. Note that healthcare professions in our Dutch sample include “well-being” (“zorg en welzijn”), which is a broader category than purely healthcare professionals. This also includes massage therapists and physiotherapist, for instance.

Moreover, although we include risk aversion (in multiple relevant domains), demographic differences, and personal exposure to COVID-19 in our analysis, we do not include personality traits. We also need to acknowledge that, although we strived to approximate personal exposure to COVID-19, we are unable to identify frontline healthcare workers that are exposed to a uniquely intense level of exposure incomparable to private life exposure. Note that Figure 2 shows that our sample holds over 15% healthcare workers, but we are unable to distinguish between frontline COVID-19 workers and healthcare workers for which exposure is comparable to other occupations (e.g., massage therapist, dentists, or physical therapists). Finally, we expect that people with a garden (or perhaps even a balcony) might find the need to recreate outdoors significantly less acute as compared to (large) families in apartments without such amenities. We specifically ask the respondents to consider a situation in which the area of recreation is the only available means of recreation, but we cannot exclude the possibility that other individual differences influence our results.

Finally, we frame our experiment as a one-shot game even though in real-life, people are able to update their information. Information about traffic jams, live news coverage of popular spots, and even witnessing crowdedness themselves once they are on the road will potentially change behavior. This includes information from past days (e.g., media coverage of previous hot days), current events (e.g., social media coverage of friends and family), and future updating (e.g., once traveling, seeing others on the streets). For some people, this information will influence their decision on the day itself, for others their commitment to their initial decision will be less easily swayed. However, we note that we do not argue that our key take-away is that all popular locations will inevitably end up crowded due to the ambiguous policy. The main result of our paper is that this policy combined with no clear and updated information of the behavior of other participants (e.g., state of the recreation spot) leads to an unintended suboptimal group decision following an (seemingly) optimal individual decision. Without correct information or information updating, this could lead to an escalation of crowdedness.

### Implications

The COVID-19 pandemic demands significant self-control from society to stay home. The recommendation to avoid crowded places creates a sense of freedom and offers the possibility to act dynamically given the circumstances. The definition of this policy advice, however, also offers freedom in interpretation. Consequently, the freedom is implicitly asking more from the population than it initially seems. “Use your common sense” is often the accompanied advice, but our results show that more and better information concerning the context is essential to make an optimal decision.

The results of this research are not predominately pessimistic. Besides the fact that the majority of respondents indicates to stay home, we also identify a strong inclination to avoid crowded places. Only after feeling that nobody stays home any longer are people legitimizing their own violation of the recommendation. Furthermore, the existing pessimism that society has regarding the behavior of others could lead to an escalation of the situation. Providing up-to-date information could be detrimental for an accurate estimation of the situation. This information could reinforce and stimulate positive behavior. Both going out as well as staying at home are rational and ethical choices. It is, however, the relevant context that determines whether going or staying leads to a rational decision, or escalation. Without this information, the outcome of a decision will remain uncertain.

Additionally, discouraging unwanted behavior should be tailored to the individuals that are more inclined to ignore the policy recommendation. Young as well as highly educated people are less sensitive to policy recommendations in the calmer contexts, and should thus be discouraged accordingly. They draw valid conclusions, but do not seem to be aware of the potential harmful consequence when a large part of society independently reasons in the same way. Here too, facilitating relevant information could offer a solution, and avoid escalation. Moreover, seeing the violations of this policy in age brackets could spark the discussion of monitoring :youth hotspots” more than other hotspots. If it would turn out that the young remain insensitive to this recommendation even after our suggested enhancement of information, differentiation in monitoring locations could be an effective detergent method policymakers should consider before relapsing to the most restricting policy to “stay at home” for all. However, at this point the interaction between punishment, monitoring, and information provision remains speculative without further examination.

Finally, the risk profile of each individual could offer a potential policy approach. Finding that the risk attitude regarding citizens’ own health plays a key role in their decision to go or stay home, suggests that campaigns emphasizing and educating people about their own health risk could improve the collective behavior of society.

## Data Availability Statement

The raw data supporting the conclusions of this article will be made available by the authors, without undue reservation, to any qualified researcher.

## Ethics Statement

The studies involving human participants were reviewed and approved by Maastricht University’s Ethical Review Committee Inner City Faculties. The patients/participants provided their written informed consent to participate in this study.

## Author Contributions

MS was responsible for the theoretical and contextual framework, questionnaire design, data collection and interpretation, statistical analysis, and writing. NK and PE were involved in data interpretation and review and editing of the manuscript. All authors contributed to the article and approved the submitted version.

## Funding

This research was self-funded by the authors.

## Conflict of Interest

The authors declare that the research was conducted in the absence of any commercial or financial relationships that could be construed as a potential conflict of interest.

## Publisher’s Note

All claims expressed in this article are solely those of the authors and do not necessarily represent those of their affiliated organizations, or those of the publisher, the editors and the reviewers. Any product that may be evaluated in this article, or claim that may be made by its manufacturer, is not guaranteed or endorsed by the publisher.

## Footnotes

^1^For an overview of the occupational division in our sample, see Figure 2.

^2^See the limitation section for the discussion of the added value of including health beliefs beyond an indicator for COVID-19 exposure.

^3^For an elaborate overview of the reliability and validation, see Dohmen et al. (2011).

^4^For an overview of the pairwise correlations, see Table 2.

^5^Note that institution trust dictates the likelihood of adherence of the population to any policy recommendation. Therefore, as background information, the second quarter of 2020 showed the highest institutional trust by the Dutch population is the last 50 quarters (Burgerperspectieven, 2020). For instance, compared to the first quarter of 2020, the trust in the government rose from 51 to 74%.

^6^The lack of significant effect of the exposure to COVID-19 could be due to a discrepancy between the official numbers and the perceived exposure by each individual. We do not suggest that the perceived exposure would be a more accurate COVID-19 exposure metric, but do acknowledge that this subject perspective (related to health beliefs) could be a relevant explanatory factor in our results on its own. See the limitation section for a further discussion on this topic.

^7^In the limitation section, we discuss some important psychological factors that could potentially explain the proportion of individuals that are not affected by the social context, and will always stay home.

^8^It is important to note that the “low context” condition has 50 to 100% more people going out compared to the other context conditions. The strength of the significance and coefficient in a logit regression is influenced by the total amount that go out in that context compared to other coefficients. However, although the significance of the effect could be more easily detected, the magnitude of the coefficient should be less affected.

^9^Note that we are explicitly not manipulating communication or policy recommendations.

## References

[ref1] Allen V. L. (1965). Situational factors In conformity. Adv. Exp. Soc. Psychol. 2, 133–175. doi: 10.1016/S0065-2601(08)60105-7

[ref2] Baumeister R. F. Leary M. R. (1995). The need to belong: desire for interpersonal attachments as a fundamental human motivation. Psychol. Bull. 117, 497–529. doi: 10.1037/0033-2909.117.3.497, PMID: 7777651

[ref3] BBC News (2020). Coronavirus: Alarm as crowds flock to European beaches.

[ref4] Braunack-Mayer A. Tooher R. Collins J. E. Street J. M. Marshall H. (2013). Understanding the school community’s response to school closures during the H1N1 2009 influenza pandemic. BMC Public Health 13:344. doi: 10.1186/1471-2458-13-344, PMID: 23587175 PMC3636092

[ref5] Brooks S. K. Webster R. K. Smith L. E. Woodland L. Wessely S. Greenberg N.. (2020). The psychological impact of quarantine and how to reduce it: rapid review of the evidence. Lancet 395, 912–920. doi: 10.1016/S0140-6736(20)30460-8, PMID: 32112714 PMC7158942

[ref6] Bugental D. B. (2000). Acquisition of the Algorithms of social life: A domain-based approach. Psychol. Bull. 126, 187–219. doi: 10.1037/0033-2909.126.2.187, PMID: 10748640

[ref7] Bults M. Beaujean D. J. M. A. De Zwart O. Kok G. Van Empelen P. Van Steenbergen J. E.. (2011). Perceived risk, anxiety, and behavioural responses of the general public during the early phase of the influenza A (H1N1) pandemic in the Netherlands: results of three consecutive online surveys. BMC Public Health 11:2. doi: 10.1186/1471-2458-11-2, PMID: 21199571 PMC3091536

[ref8] Burgerperspectieven 2020 (publicatie No. 2; Burgerperspectieven). (2020). Sociaal en Cultureel Planbureau. Available at: https://www.scp.nl/publicaties/monitors/2020/06/29/burgerperspectieven-2020---2 (Accessed July 13, 2021).

[ref9] Camerer C. F. Ho T. H. Chong J. K. (2004). A cognitive hierarchy model of games. Q. J. Econ. 119, 861–898. doi: 10.1162/0033553041502225

[ref001] Champion V. L. Skinner C. S. (2008). The health belief model. Health Behav. Health Educ.: Theory Res. Pract. 4, 45–65.

[ref10] Curley S. P. Yates J. F. Abrams R. A. (1986). Psychological sources of ambiguity avoidance. Organ. Behav. Hum. Decis. Process. 38, 230–256. doi: 10.1016/0749-5978(86)90018-X

[ref11] De Hond M. (2020). Onderzoek 4 oktober over mondkapjes en angst om besmet te worden. Available at: https://maurice.nl/2020/10/04/onderzoek-4-oktober-over-mondkapjes-en-angst-om-besmet-te-worden/ (Accessed October 4, 2020).

[ref12] Demirtaş-Madran H. A. (2021). Accepting restrictions and compliance With recommended preventive Behaviors for COVID-19: A discussion based on the key approaches and current research on fear appeals. Front. Psychol. 12:558437. doi: 10.3389/fpsyg.2021.558437, PMID: 34163389 PMC8215168

[ref13] Deutsch M. Gerard H. B. (1955). A study of normative and informational social influences upon individual judgment. J. Abnorm. Soc. Psychol. 51, 629–636. doi: 10.1037/h0046408 13286010

[ref004] Dohmen T. Falk A. Huffman D. Sunde U. Schupp J. Wagner G. (2011). Individual risk attitudes: Measurement, determinants, and behavioral consequences. Eur. Econ. Assoc. 9, 522–550.

[ref14] Falk A. Dohmen T. Huffman D. (2016). The preference survey module: A validated instrument for measuring risk, time, and social preferences. IZA Discussion Paper, No. 9674(9674).

[ref15] Festinger L. (1957). A Theory of Cognitive Dissonance, (Stanford, CA: Stanford University Press).

[ref16] Haidt J. (2001). The emotional dog and its rational tail: A social intuitionist approach to moral judgment. Psychol. Rev. 108, 814–834. doi: 10.1037/0033-295X.108.4.814, PMID: 11699120

[ref17] Hardin G. (1968). The tragedy of the commons. Science 162, 1243–1248. doi: 10.1126/science.162.3859.1243, PMID: 5699198

[ref18] Ho T. H. Su X. (2013). A dynamic level-K model in sequential games. Manag. Sci. 59, 452–469. doi: 10.1287/mnsc.1120.1645

[ref19] Holt-Lunstad J. Smith T. B. (2012). Social relationships and mortality. Soc. Personal. Psychol. Compass, 6, 41–53.

[ref20] Huremović D. (Ed.). (2019). Psychiatry of Pandemics: A Mental Health Response to Infection Outbreak. Springer, Switzerland.

[ref21] Jeong H. Yim H. W. Song Y.-J. Ki M. Min J.-A. Cho J.. (2016). Mental health status of people isolated due to Middle East respiratory syndrome. Epidemiol. Health 38:e2016048. doi: 10.4178/epih.e2016048, PMID: 28196409 PMC5177805

[ref22] Keizer K. Lindenberg S. Steg L. (2008). The spreading of disorder. Science 322, 1681–1685. doi: 10.1126/science.1161405, PMID: 19023045

[ref23] Kerr N. L. Rumble A. C. Park E. S. Ouwerkerk J. W. Parks C. D. Gallucci M.. (2009). “How many bad apples does it take to spoil the whole barrel?”: social exclusion and toleration for bad apples. J. Exp. Soc. Psychol. 45, 603–613. doi: 10.1016/j.jesp.2009.02.017

[ref24] Klein C. T. F. Helweg-Larsen M. (2002). Perceived control and the optimistic bias: A meta-analytic review. Psychol. Health 17, 437–446. doi: 10.1080/0887044022000004920

[ref25] Martínez-Marquina A. Niederle M. Vespa E. (2019). Failures in contingent reasoning: The role of uncertainty. Am. Econ. Rev. 109, 3437–3474. doi: 10.1257/aer.20171764

[ref26] Muchnik L. Aral S. Taylor S. J. (2013). Social influence bias: A randomized experiment. Science 341, 647–651. doi: 10.1126/science.1240466, PMID: 23929980

[ref002] Nash J. F. (1950). Equilibrium points in n-person games. PNAS 36, 48–49. doi: 10.1073/pnas.36.1.48 16588946 PMC1063129

[ref27] National Center for Immunization and Respiratory Diseases (NCIRD) (2020). Travel during the COVID-19 Pandemic. Available at: https://www.cdc.gov/coronavirus/2019-ncov/travelers/travel-during-covid19.html (Accessed October 15, 2020).

[ref28] Peperkoorn L. S. Becker D. V. Balliet D. Columbus S. Molho C. Van Lange P. A. M. (2020). The prevalence of dyads in social life. PLoS One 15:e0244188. doi: 10.1371/journal.pone.0244188, PMID: 33370332 PMC7769262

[ref29] Przybylski A. K. Murayama K. Dehaan C. R. Gladwell V. (2013). Motivational, emotional, and behavioral correlates of fear of missing out. Comput. Hum. Behav. 29, 1841–1848. doi: 10.1016/j.chb.2013.02.014

[ref003] RIVM (2020). Covid-19 aantallen per gemeente per publicatiedatum. Available at: https://data.rivm.nl/geonetwork/srv/dut/catalog.search#/metadata/5f6bc429-1596-490e-8618-1ed8fd768427?tab=genera

[ref30] Rutte C. G. Wilke H. A. M. (1992). “Goals, expectations and behavior in a social dilemma situation,” in Social Dilemmas: Theoretical Issues and Research Findings. 1st *Edn.* eds. Liebrand W. Messick D. Wilke H.. (Garland Science: International series in experimental social psychology), 289–305.

[ref31] Serafini G. Parmigiani B. Amerio A. Aguglia A. Sher L. Amore M. (2020). The psychological impact of COVID-19 on the mental health in the general population. QJM 113, 531–537. doi: 10.1093/qjmed/hcaa201, PMID: 32569360 PMC7337855

[ref005] Sheeran P. Webb T. L. (2016). The Intention–Behavior Gap. Soc. Personal. Psychol. Compass 10, 503–518. doi: 10.1111/spc3.12265

[ref32] Shepperd J. A. Carroll P. Grace J. Terry M. (2002). Exploring the causes of comparative optimism. Psychol. Belg. 42, 65–98. doi: 10.5334/pb.986

[ref33] Shepperd J. A. Waters E. Weinstein N. D. Klein W. M. P. (2015). A primer on unrealistic optimism. Curr. Dir. Psychol. Sci. 24, 232–237. doi: 10.1177/0963721414568341, PMID: 26089606 PMC4467896

[ref34] Stahl D. O. (1993). Evolution of Smartn players. Games Econ. Behav. 5, 604–617. doi: 10.1006/game.1993.1033

[ref35] Statistieken over het Coronavirus en COVID-19 (2021). AlleCijfers.nl. Available at: https://allecijfers.nl/nieuws/statistieken-over-het-corona-virus-en-covid19/ (Accessed April 5, 2021).

[ref36] Tyler J. M. Rosier J. G. (2009). Examining self-presentation as a motivational explanation for comparative optimism. J. Pers. Soc. Psychol. 97, 716. doi: 10.1037/a0016231, PMID: 19785488

[ref37] World Health Organization. (2020). Coronavirus disease (COVID-19) advice for the public. Available at: https://www.who.int/emergencies/diseases/novel-coronavirus-2019/advice-for-public (Accessed October 13, 2020).

